# Sexual Abuse Exposure Alters Early Processing of Emotional Words: Evidence from Event-Related Potentials

**DOI:** 10.3389/fnhum.2017.00655

**Published:** 2018-01-15

**Authors:** Laurent Grégoire, Serge Caparos, Carole-Anne Leblanc, Benoit Brisson, Isabelle Blanchette

**Affiliations:** ^1^CNAPs Lab, Department of Psychology, Louisiana State University, Baton Rouge, LA, United States; ^2^Département de Psychologie, Université de Nîmes, Nîmes, France; ^3^Département de Psychologie, Université du Québec à Trois-Rivières, Trois-Rivières, QC, Canada

**Keywords:** event-related potentials (ERPs), emotional words, Stroop, trauma, early processing

## Abstract

This study aimed to compare the time course of emotional information processing between trauma-exposed and control participants, using electrophysiological measures. We conceived an emotional Stroop task with two types of words: trauma-related emotional words and neutral words. We assessed the evoked cerebral responses of sexual abuse victims without post-traumatic stress disorder (PTSD) and no abuse participants. We focused particularly on an early wave (C1/P1), the N2pc, and the P3b. Our main result indicated an early effect (55–165 ms) of emotionality, which varied between non-exposed participants and sexual abuse victims. This suggests that potentially traumatic experiences modulate early processing of emotional information. Our findings showing neurobiological alterations in sexual abuse victims (without PTSD) suggest that exposure to highly emotional events has an important impact on neurocognitive function even in the absence of psychopathology.

## Introduction

The lifetime prevalence of experiencing any potentially traumatic event^1^ is very high (89.7% according to DSM-5 criteria; Kilpatrick et al., 2013). Exposure to traumatic situations is associated with some long-term cognitive alterations, especially in verbal memory, working memory, attention and executive functions (Jenkins et al., 1998; Galletly et al., 2001; Beers and De Bellis, 2002; Stein et al., 2002; Navalta et al., 2006). A particularly robust observation points to an effect on selective attention, characterized by an impaired attentional filtering of trauma-related emotional information in trauma-exposed individuals (e.g., Caparos and Blanchette, 2014). In this paper, we explored the electrophysiological correlates of the processing of trauma-related stimuli in victims of sexual abuse, which is a particularly widespread trauma in the general population (prevalence rates of sexual abuse range from 4.0 to 21.4% in adults and from 3.0 to 33.2% in children; Chen et al., 2010).

### Emotional stroop task with trauma victims

Trauma-exposed individuals tend to allocate more attention to stimuli related to their trauma, even when these stimuli are not task-relevant (Williams et al., 1996; Cisler et al., 2011). This outcome can notably be demonstrated using the emotional Stroop task, in which participants have to name the ink color of emotional or neutral words. The emotion-related interference (i.e., the response time difference between emotional and neutral conditions) is typically greater in trauma-exposed victims, relative to controls, when the emotional words are trauma-related. This effect was observed in individuals with or without post-traumatic stress disorder (PTSD; Cassiday et al., 1992; Cisler et al., 2011). The emotional Stroop effect in relation to trauma is relatively robust. This is confirmed by a comprehensive meta-analysis (Cisler et al., 2011), which included a consideration for unpublished studies. This work showed that the within subject difference for trauma-related vs. neutral words was *d* = 0.39 for PTSD groups and *d* = 0.24 for trauma-exposed groups. In both cases, this is more than in non-exposed control participants, and effects are significantly different from 0. More importantly, the effect sizes were robust with regard to publication bias. This meta-analysis did not specifically consider sexual abuse relative to other traumas, however analyses showed that effects sizes were larger for assault traumas (which included sexual abuse, *d* = 0.48) compared to non-assault traumas (*d* = 0.34).

While the emotional Stroop task has established that trauma exposure alters selective attention to trauma-related stimuli, it is not clear what neurocognitive processes this results from (Williams et al., 1996; Cisler et al., 2011). In particular, behavioral results obtained with this paradigm do not permit to determine the time course of processes involved in the emotional Stroop effect. The study we report here focused on this question.

Examining the neurophysiological processing of emotional information should reveal the level at which processing differences occur (e.g., early or late) between trauma-exposed victims and controls. This is important both to understand the nature of the impact of trauma on cognition, and the possibilities for altering these effects, when they are negative. Event-related potentials (ERPs) provide precise temporal data about distinct neural responses that take place during the processing of emotional and neutral information, and can be used to identify the temporal locus of this difference (Luck, 2005). A modulation of early processing is thought to be associated with perceptual, more automatic processing (Näätänen et al., 1982), while later components are generally associated with more strategic, controlled cognitive processes (Mangun and Hillyard, 1995). To date, it remains unknown whether trauma exposure affects the former or the latter.

The objective of the present study was to compare the time course of implicit^2^ processing of emotional, trauma-related words between trauma-exposed (without PTSD) and control participants, using the emotional Stroop task, which is one of the most common methods to investigate the influence of emotional content on information processing (Williams et al., 1996). We employed a modified version of the emotional Stroop paradigm, with a bilateral presentation of stimuli (a word and a string of Xs; see method section), for the needs of the electrophysiology, especially to measure the N2pc component. We only selected trauma-related words (and not general negative words) to increase the possibility of observing group differences on ERP measures. Indeed, trauma-related attentional biases tend to be specific to trauma-relevant words, rather than generally negative words (Cisler et al., 2011). Trauma-related and neutral words of our study were matched on frequency (because less frequent words cause higher interference than more frequent ones; Burt, 2002), but differed on valence and arousal dimensions, with lower valence and greater arousal for trauma-related words (see method section). Negative valence of words was shown to inflate reaction latencies in the emotional Stroop task (McKenna and Sharma, 2004). Compton et al. (2003) also pointed out that stimuli with high arousal elicited greater interference than stimuli with low arousal, and a number of studies specified that arousal effect was independent from valence (e.g., Dresler et al., 2009; Imbir, 2016). Thus, we should observe greater latencies for trauma-related words than for neutral words in the two groups of participants, with a higher effect in sexual abuse victims due to an attentional bias toward trauma-related information (Williams et al., 1996; Cisler et al., 2011). Two samples of participants were included: sexual abuse victims without PTSD and no-abuse controls. Examining trauma-exposed participants without PTSD permits to exclude PTSD symptoms as explanatory factor and to focus on effects caused by trauma exposure. We chose to focus in particular on three orthogonal ERP components, one early (C1/P1), one mid-latency (N2pc), and one late (P3b)^3^, which can be isolated with different subtraction methods (see De Beaumont et al., 2007; Kappenman and Luck, 2012). Each component is known to have a role in the attentional processing of emotional information. In this way, we planned to find out at what stage the alteration of attentional processing appears in trauma-exposed individuals performing an emotional Stroop task.

### ERP correlates of emotional information processing after trauma exposure

Few ERP studies have inspected early stages of emotional information processing in trauma-exposed victims (Javanbakht et al., 2011; Saar-Ashkenazy et al., 2015). Early visual processing of emotional stimuli can be indexed by two important components: the C1 and the P1. The C1 is detected 60–80 ms after the presentation of a visual stimulus and is thought to be related to activity of the primary visual cortex (V1). Several studies have documented a modulation of C1 by affective state (Scott et al., 2009; Eldar et al., 2010; Vanlessen et al., 2013), but not by emotional value of the stimuli. By contrast, a number of studies have shown a modulation of P1 by the emotional value of the contents, specifically in emotional Stroop tasks, with non-clinical populations. A greater amplitude of P1 was observed for emotional, particularly negative and threat-related words, compared to neutral words (Li et al., 2007; van Hooff et al., 2008; Sass et al., 2010). Altogether, these results suggest an effect of emotion on attention allocation which may occur outside the participants' control. To our knowledge, no study has yet reported a P1 or C1 effect in trauma participants with the emotional Stroop task. We examined the 55–165 ms time-window, which we have termed *early wave*, and which included the C1 and the P1.

One additional contribution of studying trauma victims, beyond understanding the consequences of trauma, is that it can refine our understanding of the neurocognitive processing of emotional value more generally. Prior studies have shown early processing modulation to emotional words, compared to neutral words. While these results seem to be related to emotion, they could also depend on other confounded characteristics of emotional words which are for instance less frequent in natural language and have smaller orthographic neighborhoods than neutral words (Larsen et al., 2006). Using the same words for trauma-exposed and non-trauma exposed individuals allowed us to ensure that the potential modulation of early ERPs is not due to confounding lexico-semantic properties of the words. In addition, it is important to examine if the effects observed in experimental studies can also be seen with more intense and personally meaningful stimuli. Such a result would indicate (1) that emotional information is indeed processed differently from neutral information at an early stage, and (2) that traumatic experiences can deeply modify the processing of trauma-related emotional information, altering processes at an early stage of processing that is less amenable to strategic processing than later processing.

Other components, occurring later in the information processing sequence, can inform us about a potential influence of traumatic experiences on more strategic, controlled processes. We were interested in testing one mid-latency (N2pc) and one latter ERP component (P3b). The N2pc is an electrophysiological index of the deployment of visual spatial attention. This component, labeled “N2pc” to designate its latency range and its occurrence at *p*osterior *c*ontralateral scalp (see e.g., Heinze et al., 1990; Luck et al., 1993; Luck and Hillyard, 1994; Eimer, 1996; Brisson et al., 2007), culminates around 200–250 ms. Fox et al. (2008) have found that individuals with high level of trait anxiety show an enhanced N2pc component for angry compared to happy faces. Likewise, Feldmann-Wüstefeld et al. (2011) have reported larger N2pc for angry compared to happy faces with a non-clinical population. These results suggest that greater attentional resources are deployed toward threatening stimuli. However, emotional processing associated to the N2pc has essentially been explored with pictures or faces (e.g., Eimer and Kiss, 2007; Buodo et al., 2010; Kappenman et al., 2015) and never, to our knowledge, with words. Moreover, no study seems to have been conducted on this component with a trauma population. Consistent with the evidence concerning an attentional bias, we hypothesized that the N2pc amplitude difference between trauma-related words and neutral words could be greater in sexual abuse victims than in non-exposed controls because trauma-related words should be more threatening for sexual abuse victims.

Another component that indicates later, more strategic processes is the P3b (which is part of the P300 complex; Polich, 2007). P3b amplitude is related to increased attentional resource deployment (e.g., Yee and Miller, 1994) and can be used as an index of updating or consolidation in short-term memory (see e.g., Bourassa et al., 2015; Brisson, 2015; but see Verleger, 1988). P3b is often larger for emotional than neutral stimuli (e.g., Schupp et al., 2004; Herbert et al., 2008), reflecting prioritization of emotional information. In what is presumably the only ERP study exploring the source of emotional Stroop interference in patients with PTSD, Metzger et al. (1997) reported greater P3b amplitude to trauma-related words compared to neutral words, but this effect did not vary as a function of group (PTSD, non-exposed healthy control). Nevertheless, the PTSD group included only nine individuals, a sample size possibly too small to observe a significant interaction between word type and group. Thomas et al. (2007) also found larger P3b amplitude for threat words than for neutral words in non-exposed healthy participants (see also, Li et al., 2007; Sass et al., 2010); supporting a preferential processing of threat. In our study, we took advantage of the well-known sensitivity of the P3b to the frequency of task-defined target category (Luck, 1998; Vogel et al., 1998; Brisson and Bourassa MÈ, 2014), by manipulating the frequency of colors, to evoke amplitude differences in order to isolate the P3b component. We expected that the processing of trauma-related words would be accentuated in trauma-exposed victims, and so they would have a greater word type effect on P3b amplitude than controls.

### Aims and hypotheses

In summary, this study aimed to examine the neurophysiological consequences of trauma exposure in relation to emotional information processing. We wanted to determine the level of processing at which differences between two groups, trauma-exposed victims (without PTSD) and no-trauma controls, occur in an emotional Stroop task, to better understand the cognitive processes responsible of these differences. We predicted to observe a greater behavioral emotional Stroop effect for victims than for control participants. Likewise, for each ERP component (early wave, N2pc and P3b), we hypothesized that amplitude differences between trauma-related words and neutral words would be greater in sexual abuse victims than in non-exposed controls. A differential word type effect between trauma-exposed victims and controls in the early wave would indicate that trauma-exposure affects more automatic processing of emotional words. A similar effect for N2pc or P3b would reveal that trauma exposure alters later, strategic processing of emotional words.

## Method

### Participants

Participants responded to poster or email ads in the community of Trois-Rivières and on campus. The study was advertised as exploring the interaction between emotions, working memory and attention. Sexual abuse was not mentioned at the recruitment stage; participants were informed that they would answer questions about past experiences of abuse during the information and consent stage. Only women were recruited since sexual abuse is more prevalent to this group (e.g., in 2008, more than 80% of sexual abuse victims were women in Canada; see http://www.statcan.gc.ca).

We determined target sample size based on effect sizes obtained in relevant prior research. In one study which is particularly relevant (Caparos and Blanchette, 2014), the effect size for the within subject difference (abuse vs. neutral words) was *d* = 0.62. A similar effect size was obtained for the between group difference comparing Stroop interferences (*d* = 0.63, emotional—neutral, in trauma exposed vs. control participants). With a goal of 0.80 power, α = 0.05, for individual *t*-test, the required sample size would be 18. The same calculation for the between group difference leads to an estimate of 32 participants required in the total sample.

Our sample included 30 participants. All participants were French native speakers and had normal or corrected-to-normal vision. Four participants were excluded because more than 50% of trials were removed (see artifact rejection criteria below) leaving a sample of 26 participants. Thirteen participants reported experiencing at least one type of sexual abuse (victims) and 13 participants reported no experience of sexual abuse (controls), as measured by the *Early Trauma Inventory Self Report-Short Form* (ETISR-SF; Bremner et al., 2007). Except sexual abuse in victims, participants reported no history of trauma. None of the participants reported having been diagnosed PTSD. We provide details about characteristics of our participants in Table 1. The study was approved by the local ethics committee of the Université du Québec à Trois-Rivières and all participants provided written informed consent prior to testing, being fully aware of the nature of the stimuli to be presented and having seen examples of the questions that would be asked. They received a financial compensation of 30$CA for their participation.

**Table 1:** Participant characteristics and test scores.

	**Victims (sexual abuse)**	**Controls (no abuse)**
Age (years)	24.08 (6.50)	20.77 (1.88)
*n*	13	13
Handedness	No left-handers	2 left-handers
ETISR-SF	2.54 (1.51)	0 (0)
STAI-S	38.15 (9.64)	35.15 (9.22)
LEI	17.46 (7.99)	13.77 (3.72)
PCL-C	34.31 (17.81)	–

*Standard deviations are in parentheses. ETISR-SF, Early Trauma Inventory Self Report-Short Form; STAI-S, State-Trait Anxiety Inventory-State; LEI, Life Events Inventory; PCL-C, Post-traumatic Stress Disorder CheckList—Civilian Version*.

#### Experimental setup

Testing took place in a dark room. The experimental protocol included the administration of several questionnaires and an emotional Stroop task during which continuous electroencephalographic activity was recorder from 64 active scalp electrodes (actiCap). Questionnaires and stimuli were presented on a CRT 17-in monitor, operating at a resolution of 1,024 × 732 pixels. Viewing distance was about 60 cm. The stimuli were generated and the experiment was run using EPrime (Schneider et al., 2002). Participants came to the laboratory for a single 90-min session.

#### Stimuli and procedure

Participants first completed a short questionnaire about demographic information (age, handedness and mother tongue). Then, they performed an adaptation of the emotional Stroop task. Finally, they evaluated the affective connotation of the Stroop words and filled in several questionnaires which assessed their level of state anxiety and their traumatic and stressful life experiences.

#### The emotional stroop task

Ten words (6 adjectives and 4 substantives^4^) were used in each of the two conditions: emotional and neutral. Emotional words were all related to sexual abuse. Each emotional word was paired with a neutral word which had the same number of letters and the same first letter (Table 2). Emotional and neutral words were matched on frequency, *t*_(18)_ = 1.12, *p* = 0.278, as derived from the online database Lexique 3.80 (http://www.lexique.org; New, 2006). We have also tested valence and arousal dimensions of words using the same method as Gilet et al. (2012). Twenty-eight participants (7 males, French native speakers, mean age 27.38 years, *SD* = 4.26), who did not participate at this study, have evaluated on line the 20 words on a 7-point scale ranging from “1 = very unpleasant, unpleasant, negative connotation” to “7 = very pleasant, pleasant, positive connotation” for valence dimension and from “1 = very calming, soothing, relief feeling” to “7 = very arousing, exciting, stressful” for arousal dimension. As expected, results revealed significantly lower valence ratings for emotional words (*M* = 1.60, *SD* = 0.62) than for neutral words (*M* = 4.25, *SD* = 0.65), *t*_(27)_ = 16.46, *p* < 0.001, *d* = 3.11, and significantly greater arousal ratings for emotional words (*M* = 5.95, *SD* = 1.05) than for neutral words (*M* = 3.63, *SD* = 0.56), *t*_(27)_ = 11.36, *p* < 0.001, *d* = 2.15 (Table 2). Each adjective was presented 54 times and each substantive was presented 27 times (adjectives were two times more frequent than substantives to observe if a semantic effect was reflected by the P3b amplitude) resulting in 432 trials per condition with a total of 864 trials. Words appeared in a random order.

**Table 2:** Valence and arousal ratings for each word of the emotional Stroop task.

**Condition**	**Grammatical class**	**Words**	**Valence**	**Arousal**
Emotional	Adjectives	abusée (abused)	1.82 (1.09)	5.57 (1.93)
		blessée (injured)	1.71 (1.18)	5.82 (1.19)
		forcée (forced)	1.57 (0.84)	5.36 (1.22)
		menacée (threatened)	1.64 (0.91)	6.11 (1.20)
		obligée (constrained)	2.29 (1.15)	5.61 (1.03)
		violée (raped)	1.11 (0.42)	6.57 (1.20)
	Substantives	abus (abuse)	1.57 (0.69)	5.79 (1.69)
		menace (threat)	1.54 (0.92)	5.93 (1.30)
		trauma (trauma)	1.61 (1.10)	6.11 (1.31)
		viol (rape)	1.11 (0.42)	6.61 (1.20)
Neutral	Adjectives	avérée (proven)	4.25 (1.32)	3.75 (1.11)
		balayée (swept)	3.54 (1.14)	4.04 (1.10)
		fermée (closed)	2.68 (1.09)	4.96 (1.50)
		moussée (foamed)	4.86 (1.24)	2.75 (1.35)
		ondulée (waved)	4.96 (1.07)	3.39 (1.40)
		voûtée (vaulted)	3.46 (1.37)	3.71 (1.21)
	Substantives	acte (act)	4.46 (1.45)	4.14 (1.04)
		mousse (foam)	5.36 (1.10)	2.36 (1.31)
		tricot (knitting)	4.50 (1.55)	2.54 (1.29)
		vite (fast)	4.14 (1.43)	4.64 (1.37)

*In the “Words” column, the English equivalent words are mentioned in parentheses. In “Valence” and “Arousal” columns, standard deviations are in parentheses*.

At the beginning of each trial, a fixation cross was displayed for 500 ms at the center of the screen. Then, two stimuli, a word and a string of Xs, were presented, one on either side of the fixation cross (spanning approximatively 4°-6.8° eccentricity), for 300 ms. When the word appeared in left visual field, the Xs appeared in right visual field and vice versa (the number of Xs was always equal to the number of letters of the word which accompanied it). This manipulation was designed to obtain the N2pc component. This component is isolated by subtracting the voltage at ipsilateral electrodes (e.g., PO8 when the target is presented in the right hemifield) form contralateral electrodes (e.g., PO7 when the target is presented in the right hemifield; Luck and Hillyard, 1994). The side of presentation of words was randomized, but in each condition, words (and strings of Xs) appeared as frequently on the left side as on the right side of the screen.

Given that the N2pc component is larger in amplitude for stimuli presented in the lower visual field (compared to stimuli presented in the upper visual field; Luck et al., 1997; Perron et al., 2009), stimuli were presented 0.6° below the central fixation cross, in order to increase the probability of observing an N2pc.

One of the two stimuli (the word or the Xs) was colored in each trial. Four colors were used: green, blue, orange and purple. The frequency of appearance of these four colors was manipulated in order to measure the P3b component, which is evoked to infrequent stimuli (Polich and Kok, 1995). Thus, in each condition, the blue and orange colors appeared 162 times each (75% of trials), while the green and purple colors appeared only 54 times each (25% of trials). Amplitude of the P3b is larger to less frequent stimuli, in this case the colors green and purple. Therefore, subtracting frequent trials (i.e., with blue or orange colors) from infrequent trials (i.e., with green or purple colors) isolates the P3b from overlapping activity that is not sensitive to our category-defined frequency manipulation. The order of presentation of colors was randomized.

The colored item could be either the word or the string of Xs. The purpose of this manipulation was to avoid participants focusing their attention only on words. Thus, we wanted to exclude the possibility that participants would use the lexical information to detect the color more quickly. Indeed, if participants had noted that color was always on words, the lexical information could have been eventually used to process the color faster. In each condition, the colored item was the word 384 of the times, while it was the strings of Xs 48 of the times, in a random order (among the 48 trials in which the color appeared on the Xs, each adjective was presented 6 times and each noun was presented 3 times). The analyses reported in this paper were performed on trials in which color appeared on words.

Words and strings of Xs appeared in uppercase bold, font “Courrier New 13.” Fixation cross and stimuli (except the colored stimuli) were printed in black over a white background on the computer screen.

**Figure 1:**
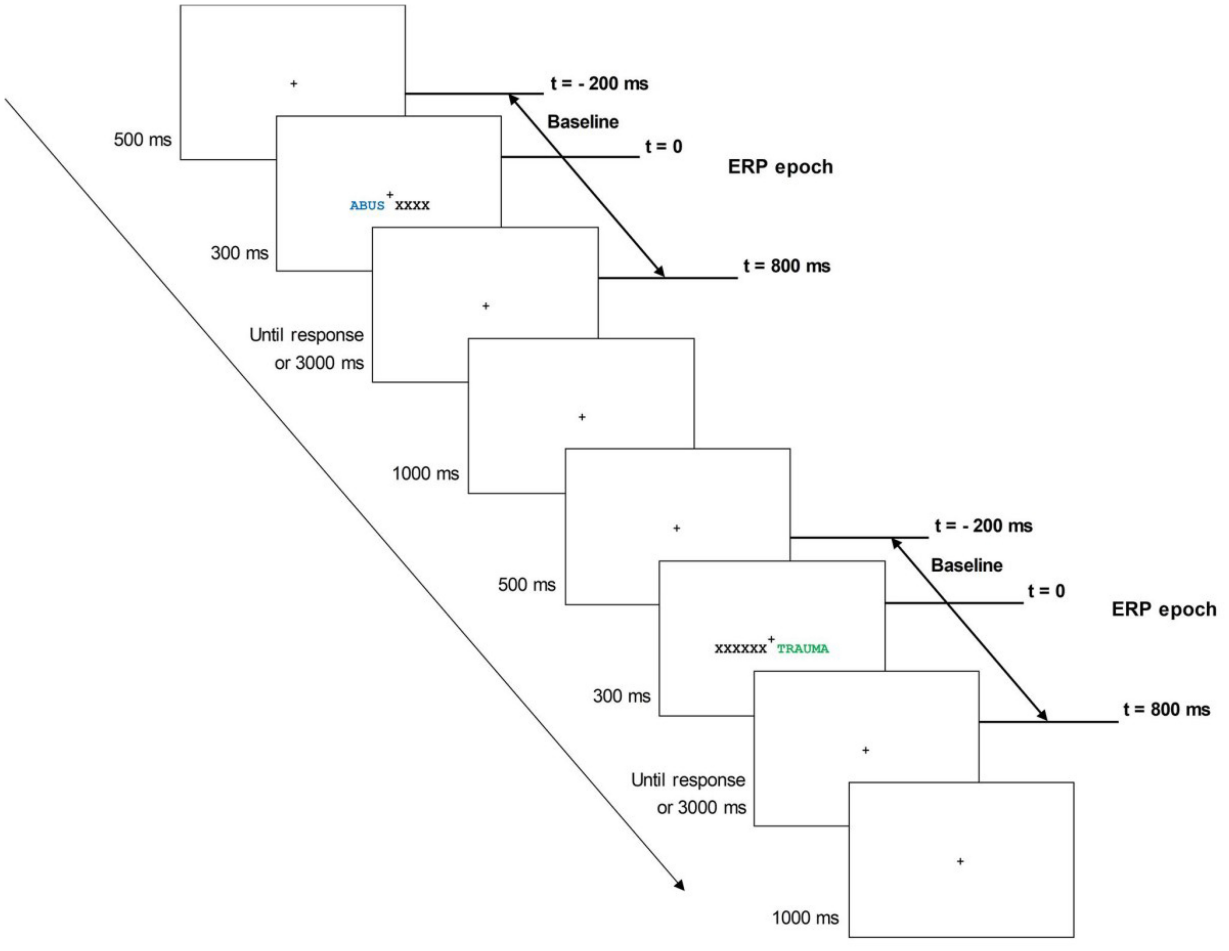
Sequence of events in the emotional Stroop task. Each trial began with a fixation cross of 500 ms, followed by a bilateral presentation of experimental stimuli for 300 ms. Then, the participant had to indicate manually the color of the colored item. After participants had responded, or after 3,000 ms had elapsed, a 1,000 ms wait followed and the next trial started.

The two stimuli (word and string of Xs) were presented bilaterally for 300 ms, after which participants had to indicate manually the color of the colored item. Participants had to press the “X” key when the colored item was presented in green or orange, and they had to press the “M” key when the colored item was presented in blue or purple. Participants were instructed to keep the index of the left hand above the “X” key and the index of the right hand above the “M” key throughout the Stroop task. They were also encouraged to respond as fast and as accurately as possible while ignoring the word's meaning. After participants had responded, or after 3,000 ms had elapsed, a 1,000 ms wait followed and the next trial started (Figure 1). All along the emotional Stroop task, the fixation cross remained at the center of the screen and participants had to maintain fixation on it to avoid eye movements or blinks during trials. If they needed to blink, participants were instructed to do so after pressing the response key.

Emotional and neutral stimuli were presented in a blocked fashion, with random order of word presentation within each block, because this method is known to potentiate the emotional Stroop effect (e.g., Kaspi et al., 1995; see also, Algom et al., 2004, Experiment 6; Holle et al., 1997). The order of presentation of conditions was counterbalanced across participants. There were 36 blocks of 12 trials in each condition with a self-paced break between blocks, to allow participants to rest, move, or blink. Before the start of the emotional Stroop task, participants performed eight practice trials (with four neutral words, different from the experimental words, repeated twice).

#### Emotionality ratings

After completing the emotional Stroop task, participants were required to evaluate the affective connotation of the 20 Stroop words. Words were rated on a 5-point scale ranging from “1 = neutral” to “5 = emotional.” Each word was presented individually at the center of the screen, in a random order, until participants had rated it.

#### Questionnaires

Participants then completed several questionnaires: four for the victims, three for the controls.

A French version of the *State-Trait Anxiety Inventory* (STAI; Gauthier and Bouchard, 1993) was filled by participants to evaluate their situational anxiety level (only form Y-1 of the test, assessing state anxiety, was presented). Participants had to choose, for each of twenty items (e.g., “I am a steady person”), an answer ranging from “1: Not at all” to “4: Very much” corresponding to their current situation. This questionnaire provides a score from 20 to 80 with higher scores indicating greater levels of state anxiety.

The subsequent questionnaire was a French version of the *Life Events Inventory* (LEI; Cochrane and Robertson, 1973). It was administered to evaluate the occurrence of 52 stressful life events (pleasant, unpleasant or neutral; for example moving to a new house, pregnancy, divorce). Participants simply indicated whether each event had occurred to them or not. Answers were summed with higher scores indicating greater numbers of stressful life events.

Finally, participants completed the sexual abuse subscale of the French version of the ETISR-SF (Bremner et al., 2007) which screens for the occurrence of traumatic sexual event(s). Participants were asked whether they had ever (1) been touched against their will on intimate parts of their body, (2) been rubbed by someone else's private parts against their will, (3) been forced to touch the intimate parts of someone else's body, (4) been forced to take part in a genital sexual intercourse, (5) been forced to take part in an oral sexual intercourse, and/or (6) been forced to kiss someone in a sexual way. Participants were considered in the victim group if they reported having experienced at least one of the items excluding the first which does not meet the legal definition for sexual abuse in the province of Quebec where the study was conducted. Victims filled a second questionnaire, namely, a French-translated version of the Post-traumatic Stress Disorder CheckList—Civilian Version (PCL-C; Blanchard et al., 1996). This 17-item questionnaire indexed the incidence of post-traumatic stress symptoms related to the traumatic sexual event(s). Participants gave an answer from one (not at all) to five (extremely) to each item. Items addressed symptoms often associated with PTSD, such as the occurrence of disturbing dreams, the inability to concentrate or the intrusion of disturbing thoughts related to the event. Answers were summed to produce a score from 17 (no post-traumatic stress symptoms) to 85 (maximum level of post-traumatic stress symptoms). The PCL-C questionnaire was used to observe whether the level of PTSD symptoms correlated with the emotional Stroop effect, even if our goal was not to study a clinical population.

#### Electrophysiological data acquisition

The electroencephalography (EEG) was recorded from 64 active Ag/AgCl electrodes (ActiCHamp system with ActiCAP, Brain Products) positioned according to the standard 10–10 system, with the exception that the TP9 and TP10 electrode sites were not used; they were replaced by electrodes placed at the mastoids. Activity at all electrodes was recorded with a left-mastoid reference, and the data were re-referenced offline to the algebraic average of the left and right mastoids (Luck, 2005). Additional cutaneous electrodes were used to monitor electrooculographic activity; two placed on external canthi to record the horizontal electrooculogram (HEOG) and two placed on infra/supraorbital regions of the right eye to record the vertical electrooculogram (VEOG). All electrode impedances were kept below 15 kΩ. The EEG was sampled and digitized at 500 Hz.

#### Event-related potentials analysis

Using the software Brain Vision Analyser 2.0 (Brain Products, Germany), signals were high-pass filtered at 0.01 Hz and low-passed at 40 Hz offline. Trials with eye blinks (VEOG > 80 μV), large horizontal eye movements (HEOG > 35 μV), and/or other artifacts at electrodes of interest (>80 μV at PO7, PO8, POz or POz) were excluded from further analyses using an automated screening procedure. The EEG was segmented relative to the onset of the presentation of each stimulus in the emotional Stroop task to create stimulus-locked epochs of 1,000 ms that included a 200 ms pre-stimulus period, which served as the epoch baseline (Figure 1). For each ERP component, epochs were averaged after removing error trials and trials with response times (RTs) beyond 1,200 ms and below 300 ms.

The mean amplitude of C1/P1 complex (time window: 55–165 ms post-stimulus) was computed separately for emotional and neutral conditions at PO7 and PO8 electrode sites, where the deflection is typically maximal.

The N2pc's mean amplitude (time window: 170–270 ms post-stimulus) was also computed at PO7 and PO8 electrode sites, were the component is maximal. To isolate the N2pc component, epochs were averaged separately for trials when the word appeared in the right visual field and trials when the word appeared in the left visual field. The N2pc component was obtained by subtracting right word trials from left word trials. The N2pc was therefore negative at PO7 (which is contralateral to words presented in the right visual hemifield and ipsilateral to words presented in the left visual hemifield) and positive at PO8 (which is ipsilateral to words presented in the right visual hemifield and contralateral to words presented in the left visual hemifield). The polarity of the PO8 electrode site was inversed for statistical analyses. This method was applied for emotional and neutral words separately leading to an N2pc component for each of these two conditions and for each of the two electrodes (PO7, left hemisphere and PO8, right hemisphere).

The mean amplitude of the P3b (time window: 450–800 ms post-stimulus) was computed separately for emotional and neutral words at the POz electrode site, where the component is typically maximal. To isolate the P3b component, we subtracted the average signals from trials with the frequent colors (blue and orange) from the average of trials with infrequent color (green and purple).

### Statistical data analysis

As mentioned previously, Stroop analyses (behavioral and electrophysiological) were conducted only with trials in which the colored item was the word (*n* = 384 per condition). In the emotional Stroop task, trials with errors (3.96%) and correct trials with RTs under 300 ms or over 1,200 ms (3.80%) were discarded from RT as well as ERP analyses. RTs, accuracy (error rates) and mean ERP amplitudes at the selected electrodes (separate for each component) were analyzed using repeated measures ANOVA with Condition (emotional, neutral) as a within-subject variable and Group (victims, control) as a between-subject variable. We added laterality (left or right hemisphere electrode, respectively, PO7 and PO8) as within-subject variable into the ANOVA to analyse C1/P1 and N2pc. Correlations between RTs as well as ERP amplitudes with the different questionnaire scores were also examined. We verified homogeneity of variances with the Levene's test (Fox, 1997). When this test suggested inequality of variances, we applied the necessary corrections (i.e., degrees of freedom were adjusted).

## Results

### Questionnaires

Average STAI-S and LEI scores are presented in Table 1. STAI-S scores were not significantly different between victims and controls, *t*_(25)_ = 0.81, *p* = 0.425, indicating the two groups did not differ in their level of situational anxiety. LEI scores were also not significantly different between victims and controls, *t*_(16.98)_ = 1.51, *p* = 0.149. Victims on average did not report a greater number of stressful life events than control participants.

### Emotionality ratings

Emotionality ratings are presented in Table 3. As expected, subjective emotionality ratings were higher for emotional words than for neutral words, *t*_(25)_ = 15.11, *p* < 0.001, *d* = 2.96. We observed a marginally significant effect of Group, *t*_(24)_ = 1.92, *p* = 0.067, *d* = 0.38 Victims rated words as overall more emotional than control participants. However, no interaction between Condition and Group was observed, *t*_(18.56)_ = 0.50, *p* = 0.626. Thus, victims did not rate the abuse-related words as particularly emotional, compared to the control group.

**Table 3:** Emotionality ratings as a function of Condition (emotional, neutral) and Group (victims, controls).

	**Victims**	**Controls**
Emotional	4.09 (0.70)	3.60 (1.13)
Neutral	1.53 (0.33)	1.20 (0.20)

*Standard deviations are in parentheses*.

### Behavioral results

Concerning errors rates, no significant main effect of Group was observed, *F*_(1, 24)_ = 0.90, *p* = 0.351. The rate of errors was not higher for victims (*M* = 3.59%, *SD* = 2.17) than for control participants (*M* = 4.33%, *SD* = 1.89). There was also neither a significant main effect of Condition, *F*_(1, 24)_ = 0.04, *p* = 0.846, nor a Condition x Group interaction, *F*_(1, 24)_ = 0.04, *p* = 0.850.

For RTs, there were no significant main effects of group, *F*_(1, 24)_ = 0.32, *p* = 0.579, or condition, *F*_(1, 24)_ = 0.12, *p* = 0.729, and no Condition x Group interaction, *F*_(1, 24)_ = 0.04, *p* = 0.846. RT values are presented in Table 4.

**Table 4:** RT (in ms) values in the emotional Stroop task as a function of Condition (emotional, neutral) and Group (victims, controls).

	**Victims**	**Controls**
Emotional	574.88 (90.45)	552.97 (96.10)
Neutral	568.93 (96.08)	551.31 (92.77)

*Standard deviations are in parentheses*.

### Electrophysiological results

The mean percentage of trials retained after rejecting artifacts was 78% in the neutral condition (minimum = 54%, maximum = 94%) and 80% in the emotional condition (minimum = 58%, maximum = 91%).

#### Early wave (55–165 ms)

**Figure 2:**
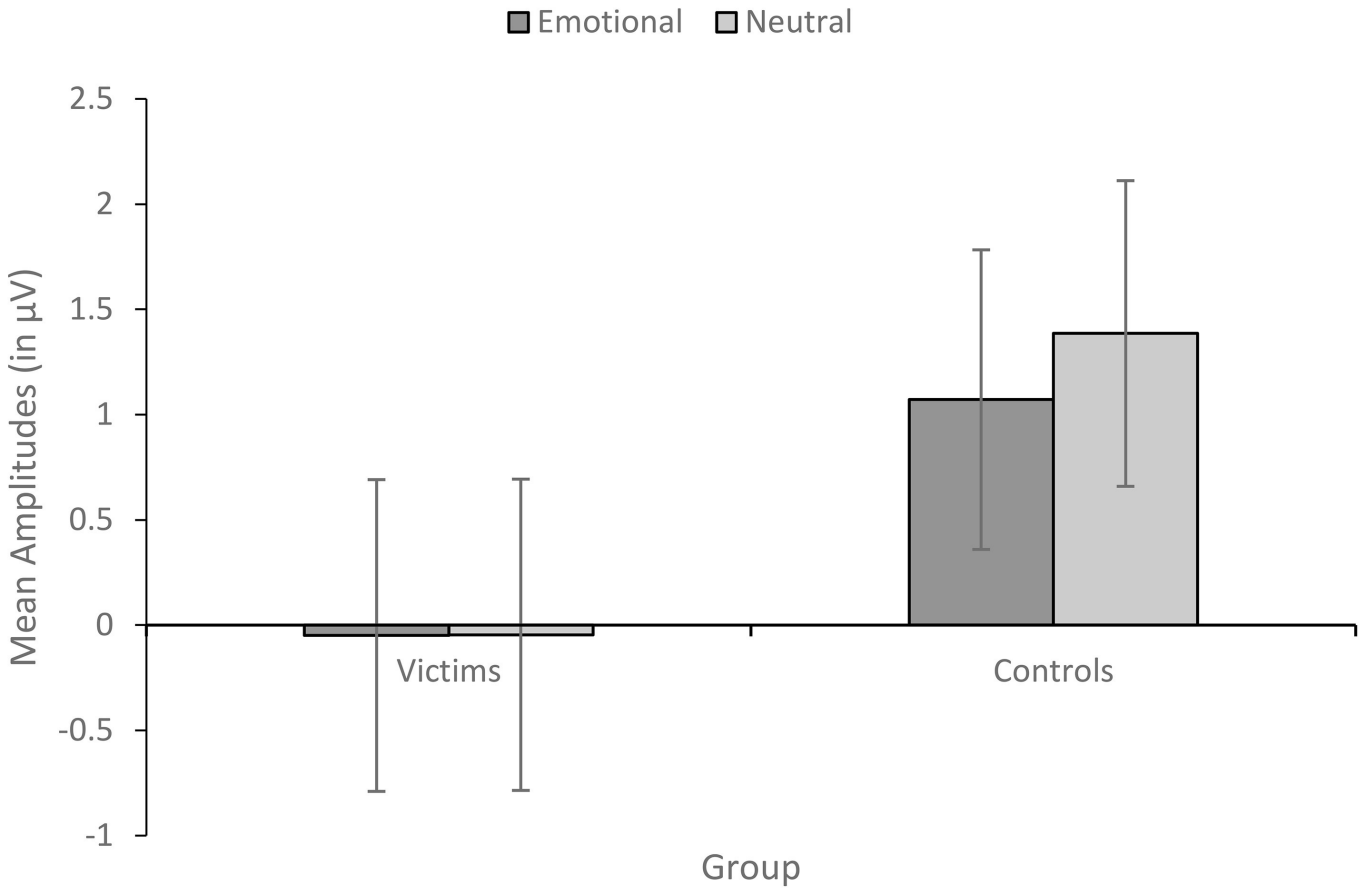
Mean amplitudes for early wave (55–165 ms) as a function of condition and group. Error bars indicate standard errors.

**Figure 3:**
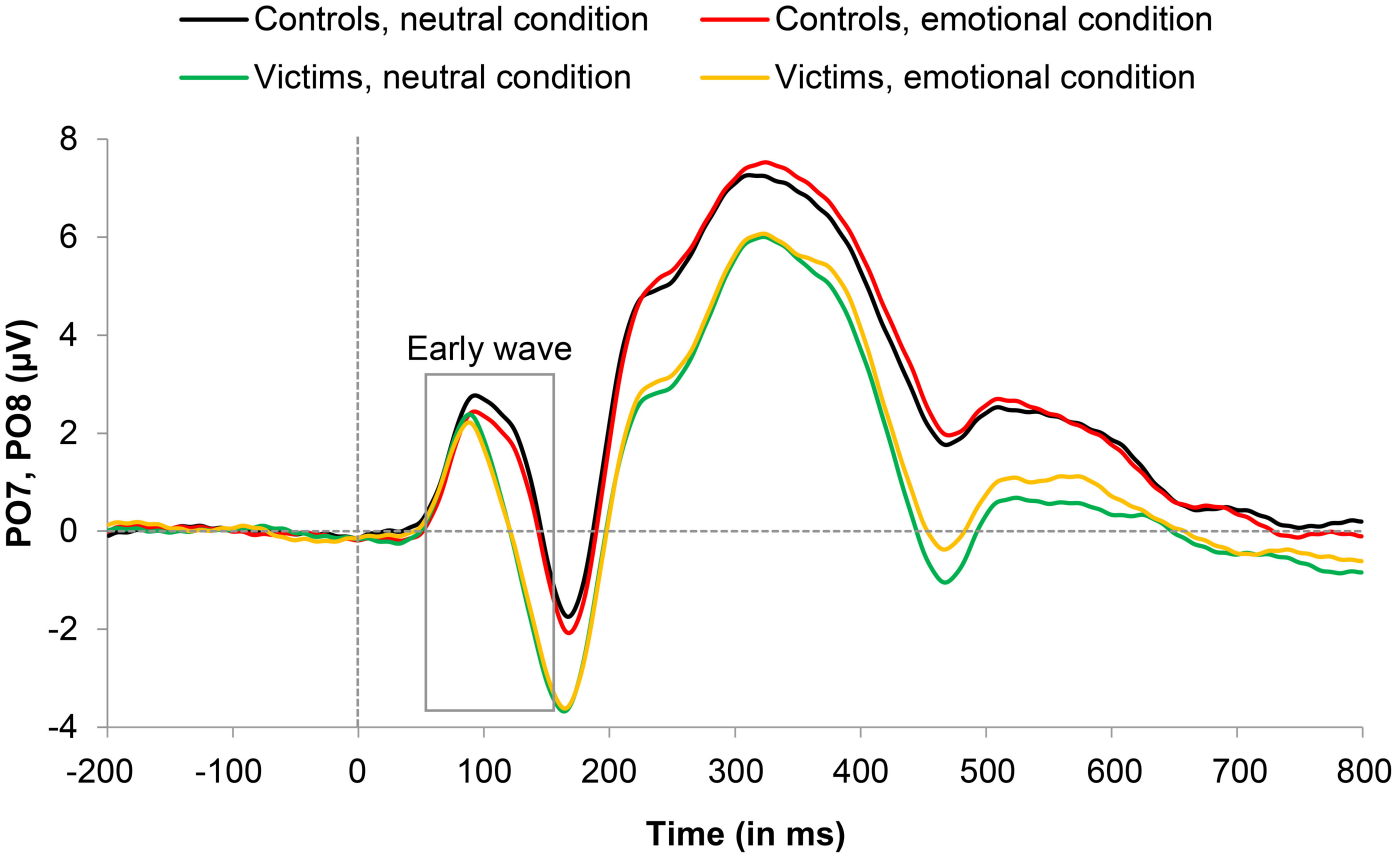
Grand average event-related potential (ERP) waveforms representing the early wave (55–165 ms) component for victims and controls in emotional and neutral conditions. Artifact-free trials with a correct response were included in the grand-average ERPs (see text for details).

C1/P1 amplitude showed a significant main effect of Condition, *F*_(1, 24)_ = 4.47, *p* = 0.045, $\eta_{p}^{2}$ = 0.157, with greater amplitude for neutral than emotional words. Crucially, this last difference was significantly larger in control participants than in sexual abuse victims as revealed by the interaction between Condition and Group, *F*_(1, 24)_ = 4.27, *p* = 0.049, $\eta_{p}^{2}$ = 0.151. Subsequent *t*-tests revealed that neutral words produced significantly higher amplitudes compared to emotional words in control participants, *t*_(12)_ = 3.10, *p* = 0.009, *d* = 0.12, but not in victims, *t*_(12)_ = 0.03, *p* = 0.976 (Figure 2). All other effects were no significant. We observed no main effect of Group, *F*_(1, 24)_ = 1.54, *p* = 0.227, or Laterality, *F*_(1, 24)_ = 0.56, *p* = 0.462, no Group x Laterality interaction, *F*_(1, 24)_ = 0.50, *p* = 0.826, no Condition x Laterality interaction, *F*_(1, 24)_ = 0.36, *p* = 0.555, and no Condition x Laterality x Group, *F*_(1, 24)_ = 0.01, *p* = 0.938. Grand-average ERP waveforms for the early wave are illustrated as a function of condition and group in Figure 3.

#### N2pc (170–270 ms)

**Figure 4:**
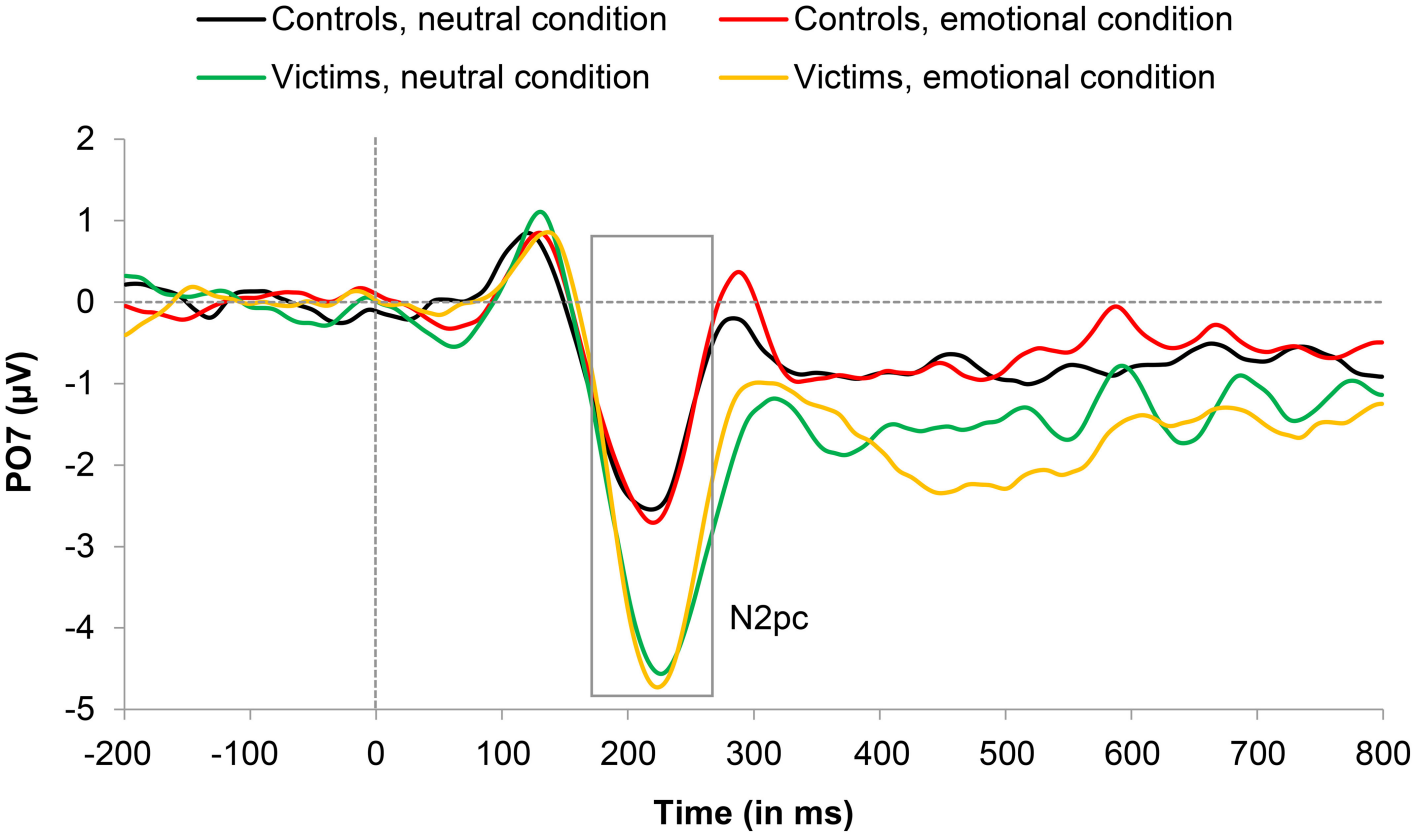
Grand average event-related potential (ERP) waveforms representing the N2pc component (170–270 ms) for victims and controls in emotional and neutral conditions. Artifact-free trials with a correct response were included in the grand-average ERPs (see text for details).

N2pc amplitude showed no main effect of Condition, *F*_(1, 24)_ = 0.10, *p* = 0.922, and no interaction between Condition and Group, *F*_(1, 24)_ = 0.84, *p* = 0.368, but a significant Group × Laterality interaction, *F*_(1, 24)_ = 5.64, *p* = 0.026, $\eta_{p}^{2}$ = 0.190. Subsequent *t*-tests revealed that victims produced significantly larger amplitudes than controls at PO7 electrode (left hemisphere), *t*_(24)_ = 2.21, *p* = 0.037, *d* = 0.87, but no group effect was observed at PO8 electrode (right hemisphere), *t*_(24)_ = 0.38, *p* = 0.709. All other effects were not significant. We observed no main effect of Group, *F*_(1, 24)_ = 1.36, *p* = 0.255, or Laterality, *F*_(1, 24)_ = 0.21, *p* = 0.654, no Condition x Laterality interaction, *F*_(1, 24)_ = 0.01, *p* = 0.940, and no Condition x Laterality x Group, *F*_(1, 24)_ = 0.10, *p* = 0.759. Grand-average ERP waveforms for the N2pc are illustrated as a function of condition and group (for the PO7 electrode) in Figure 4.

#### P3b (450–800 ms)

**Figure 5:**
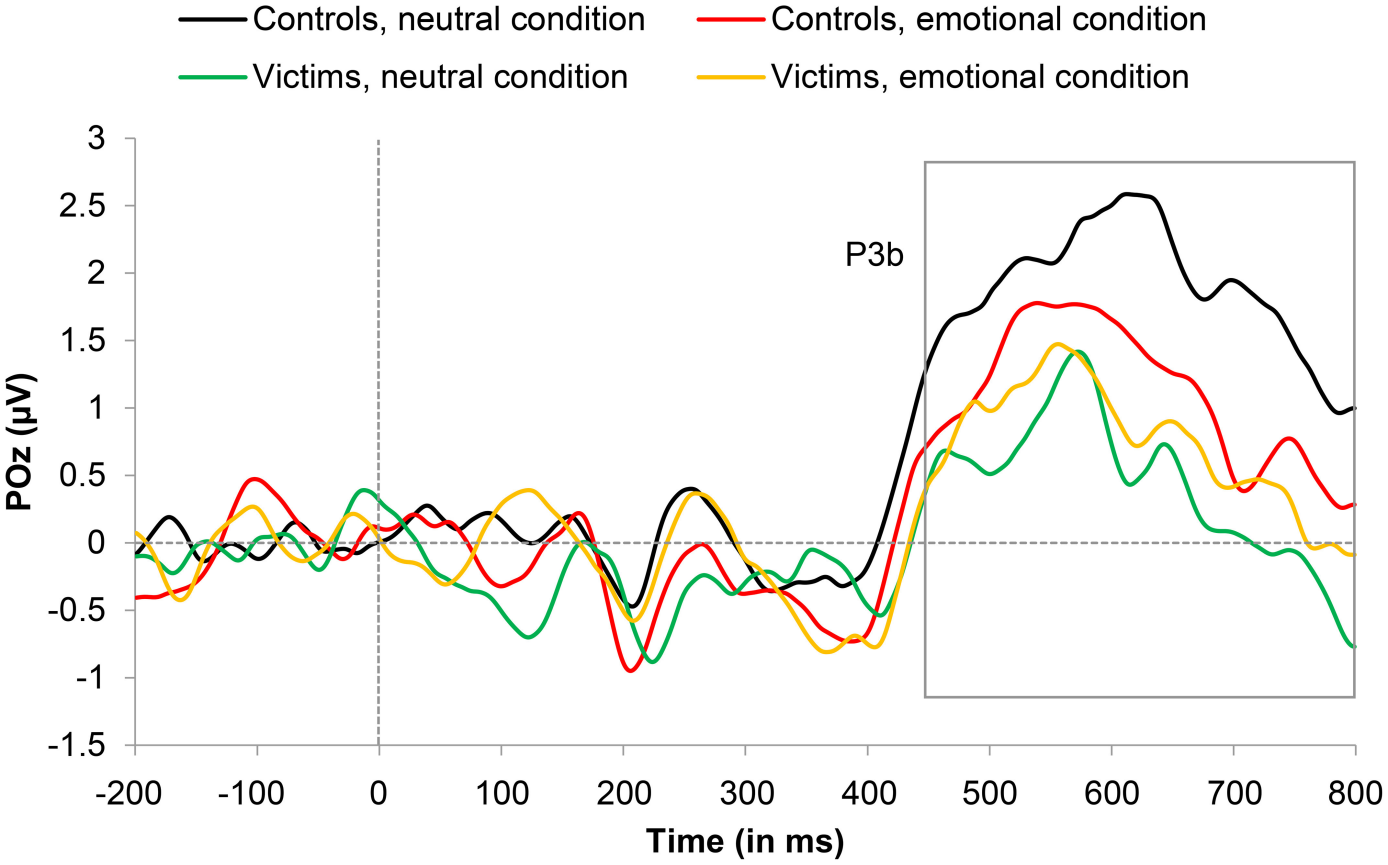
Grand average event-related potential (ERP) waveforms representing the P3b component (450–800 ms) for victims and controls in emotional and neutral conditions. Artifact-free trials with a correct response were included in the grand-average ERPs (see text for details).

P3b amplitude showed no main effect of Condition, *F*_(1, 24)_ = 0.56, *p* = 0.460, but a marginally significant effect of Group, *F*_(1, 24)_ = 4.17, *p* = 0.052, $\eta_{p}^{2}$ = 0.148, with greater amplitude in controls than in victims. This last difference was greater for neutral words than for emotional words, as indicated by a marginally significant Condition x Group interaction, *F*_(1, 24)_ = 2.98, *p* = 0.097, $\eta_{p}^{2}$ = 0.110. Subsequent *t*-tests revealed a significant group effect for neutral words, *t*_(24)_ = 2.56, *p* = 0.019, *d* = 0.99, but not for emotional words, *t*_(24)_ = 0.49, *p* = 0.707. Grand-average ERP waveforms for the P3b are illustrated as a function of condition and group in Figure 5.

### Correlational analyses

The level of interference in the emotional Stroop task (mean RTs for emotional words—mean RTs for neutral words) was not correlated with STAI-S score, *r*_(24)_ = −0.066, *p* = 0.748, LEI score, *r*_(24)_ = −0.091, *p* = 0.658, ETISR-SF score, *r*_(24)_ = 0.177, *p* = 0.563, and PCL-C score (for victims only), *r*_(11)_ = 0.374, *p* = 0.209.

The amplitude difference for the early wave in the emotional Stroop task (mean amplitude for emotional words—mean amplitude for neutral words) was not correlated with STAI-S score, *r*_(24)_ = 0.327, *p* = 0.102, LEI score, *r*_(24)_ = 0.133, *p* = 0.516, ETISR-SF score, *r*_(24)_ = 0.341, *p* = 0.089, and PCL-C score (for victims only), *r*_(11)_ = 0.119, *p* = 0.699.

## Discussion

This study aimed to determine the time course of emotional information processing in sexual abuse victims without PTSD using the Stroop paradigm. The striking result of our experiment was an early effect (55–165 ms) of emotionality which varied as a function of group (sexual abuse victims and non-exposed controls). This suggests that early, possible implicit non-strategic aspects of information processing can be altered by personal emotional experiences. Differences between groups as a function of the emotionality of stimuli for later components, the N2pc and P3b were either not observed or less marked.

### Are there ERP evidences of differential processing between trauma-exposed and control participants in the emotional stroop task?

Our results confirm the findings of previous studies showing that word processing can be affected by emotional valence very early (Li et al., 2007; van Hooff et al., 2008; Sass et al., 2010). For participants in the control group, before 165 ms following word presentation, the amplitude at occipital electrodes varied depending on emotional value. Importantly, our results show that trauma-exposure alters this early effect. This is the case even when participants do not develop significant levels of PTSD symptoms, such as was the case generally for the participants in our sample. Participants in the trauma-exposed and control groups evaluated the trauma-related stimuli as similarly emotional. Despite similar explicit emotional evaluations, the trauma-exposed participants showed an attenuation of the early electrophysiological response to the emotional stimuli.

One unexpected aspect of our results was that neutral words elicited larger early wave amplitudes than trauma-related emotional words. We had expected the opposite effect (i.e., greater amplitudes for emotional than neutral words). The explanation for this reversed effect remains to be elucidated, and will be the point of future studies, though it does not detract from our main finding. Our study is not the only one to have found this pattern of difference. Scott et al. (2009) for example, found an attenuated P1 in response to negative compared to neutral (and positive) words. This was particularly marked for high frequency words. While the researchers do not propose a complete explanation for this finding, they suggest it may be explained with reference to an early negative going wave which would be specifically elicited by the processing of negative emotional stimuli and linked with attentional prioritization. In addition to this, we also found that the difference between emotional and neutral stimuli was attenuated in trauma-exposed participants, while we had expected it would have been increased. There are however different lines of evidence suggesting blunted physiological responses to stressful stimuli in relation to trauma-exposure, particularly in relation to hormonal and autonomic responses (Depierro et al., 2013; Schalinski et al., 2015).

Although this interpretation is somewhat speculative, the main result of the present study, the fact that the early effect of emotional value on electrophysiological processing differed between groups, shows that exposition to a traumatic event can elicit long-term alterations of the implicit processing of trauma-related emotional words. This early effect that we identified may be related to categorical emotion processing (trauma- or fear-related), or the high arousal value of the trauma-related words, rather than general valence (negative), following recent electrophysiological evidence for the time course of these two types of processing (Hofmann et al., 2009; Briesemeister et al., 2014; Imbir et al., 2017). Nevertheless, emotional (trauma-related) words used in this study had lower valence and higher arousal ratings than neutral words, so we cannot determine which dimension (valence or arousal) accounts for effects observed on early wave.

It must be stressed that the alteration of the early processing of emotional words observed in sexual abuse victims was related to traumatic exposition, irrespective of PTSD^5^, whereas prior studies which have revealed an early emotional processing with trauma-exposed participants focused almost exclusively on PSTD. In an oddball paradigm, Blomhoff et al. (1998) observed a significant relationship between early neural responses (50–150 ms) to emotional words and post-traumatic stress symptoms, in particular avoidance and intrusion. Using a similar task, Attias et al. (1996) found that combat-related pictures elicited enhanced N1 amplitudes in combat veterans with PTSD compared to controls (see also, Ehlers et al., 2006). All of these studies have focused on PTSD symptoms, rather than on trauma exposure. More recently, Zhang et al. (2014) reported an interaction between emotional content (trauma-related and neutral words) and group (earthquake survivors without PTSD and control participants) on the amplitude of the P1, using a modified version of the dot probe paradigm. Earthquake-exposed survivors showed a larger P1 amplitude than the controls on congruent trials (i.e., when the trauma-related word and the target appeared on the same side), suggesting an enhanced allocation of attention to trauma-related stimuli in earthquake-exposed survivors. These authors concluded that functional brain alterations can occur in trauma-exposed survivors even if they are not presenting PTSD symptoms. Our data confirms the presence of neurobiological alterations in trauma-exposed victims without PTSD.

We also observed an absence of correlation between PCL-C score and the amplitude difference for the early wave in the emotional Stroop task (mean amplitude for emotional words—mean amplitude for neutral words). Thus, there was no indication of a relation between symptom severity and early processing of emotional words in victims (though this needs to be interpreted with caution, given the small number of participants).

Importantly, the difference between groups in the early wave cannot be explained by the subjective ratings of emotionality of Stroop words because the abuse-related words were not judged as being more or less emotional by the abuse victims, excluding the possibility that this variable was responsible for the interaction observed on the early wave. The two groups of participants were also similar in terms of state anxiety. Altogether, this suggests that the early, possibly automatic processing of abuse-related information was affected by participants' previous life experiences, independently of psychopathological symptoms or characteristics of the words.

The N2pc and P3b components (e.g., Luck, 2005) for trauma-related and neutral words did not differ nor did this interact with trauma exposure, though there was a marginally significant interaction in the case of the P3b. We noted that the P3b data were very noisy (see Figure 5). This could stem from the bilateral presentation of stimuli (used to obtain the N2pc component) which is unusual in an emotional Stroop task. Participants had to focus their attention on the colored item (inhibiting the verbal information) and ignore the non-colored item to respond efficiently. The attentional effort necessary to process the relevant information and repress the irrelevant ones might have disturbed the P3b measures. Though it is difficult to draw strong conclusions based on these null and marginally significant findings, we can at least conclude that the effect of trauma-related information, in our study, was stronger for early than for late components.

### The absence of behavioral emotional stroop effect

No behavioral effect was obtained in the emotional Stroop task. We observed no difference on RTs between emotional and neutral words for victims or controls, and no differences between groups. Although not expected, this result was coherent with the absence of electrophysiological effect on N2pc and P3b components which are associated with more explicit, strategic processing. Previous studies have shown longer RTs in the emotional condition relative to the neutral condition with sexual abuse victims, suggesting an impaired attentional filtering of trauma-related emotional information in trauma-exposed individuals (Foa et al., 1991; Cassiday et al., 1992; Caparos and Blanchette, 2014). However, the emotional Stroop effect may be absent in some conditions. Indeed, Sharma and McKenna (2001) have reported that this effect tends to decline when the interstimulus interval (ISI) increase. Numerous studies have also observed no effect when ISI was greater or equal to 1,500 ms (e.g., Frühholz et al., 2011, with an ISI of 1,500 ms; McNeely et al., 2008, with an ISI of 3,000 ms; Sass et al., 2010, with an ISI of 2,000 ± 225 ms; Thomas et al., 2007, with an ISI varying randomly between 2,500 and 3,500 ms; Zurrón et al., 2013, with an ISI of 2,500 ms). We have chosen to use an ISI of 1,500 ms in the present study for the needs of the electrophysiology (notably to enable participants to blink between two trials). This parameter might have led to the absence of the emotional Stroop effect in RTs but the electrophysiological results were our primary interest. The modified version of the emotional Stroop task could also have attenuated the behavioral Stroop effect. In our situation, two stimuli, a word and a string of Xs, were bilaterally presented in order to measure the N2pc component. Kahneman and Chajczyk (1983) showed that Stroop interference, observed when participants named the color of a patch above or below an incongruent color-word printed in black, diminished if a string of Xs was presented on the opposite side of the color-word. Thus, the bilateral presentation employed in the present study might have decreased the emotional Stroop effect. Moreover, the number of words used in our experiment (10 in each condition) was not very large, and in consequence, words were frequently repeated. Ben-Haim et al. (2014) showed that the repeated presentation of the same small set of negative words attenuates the behavioral emotional Stroop effect (because of habituation). It is also possible that effects observed on ERP components, especially N2pc and P3b, which reflects strategic, controlled cognitive processes, were attenuated by a habituation effect. Future investigations should use a larger set of words to avoid this potential problem.

The lack of difference between groups on the behavioral task or on some of the evoked potentials may be related to our small sample size. We had a target sample size of 32, knowing that 18 per group would have been ideal. Our actual sample was slightly short of this, but not by much. It must be noted that our sample size favorably compares to similar prior work using electrophysiological measures of trauma or PTSD populations, which have generally included fewer participants (see Appendix). While statistical power is highly desirable, it has to be balanced with the difficulties of accessing specific populations in sensitive situations. Research with these samples is nevertheless important. It is also important to note that our sample size was sufficient to observe a statistically significant effect on the amplitude of the early wave and this allows us to conclude that while we might have failed to detect other effects (later effects, involving more strategic processes), we can nevertheless conclude that in this context, early perceptual effects were more predominant.

## Conclusion

In summary, the major contribution of this paper was to show an alteration of early implicit processing of trauma-related emotional words in sexual abuse victims. This suggests that highly negative, potentially traumatic life events can affect automatic mechanisms involved in personally-relevant emotional information processing.

## Author contributions

LG contributed to this manuscript, including conducting literature review, participant testing sessions, EEG and behavioral analysis and the preparation of this manuscript. SC contributed by conducting literature review and creating the experimental design. C-AL contributed to this manuscript, including participant testing sessions and EEG analysis. BB contributed by analyzing EEG data and supervising the manuscript editing. IB contributed by supervising the experimental design and the manuscript editing.

### Conflict of interest statement

The authors declare that the research was conducted in the absence of any commercial or financial relationships that could be construed as a potential conflict of interest.

## Footnotes

^1^We specified *potentially* because the perception of a harrowing situation is subjective. The same event may be traumatic for an individual and not for another. In the rest of this paper, we did not repeat the term *potentially* when referring to potentially traumatic situations only to lighten the text but it is implied.

^2^By which we mean unintentional in this paper.

^3^We also planned to focus on the early posterior negativity (EPN), which is sensitive to emotional processing, but we found no effect on this component, so we did not mention EPN analyses for reasons of conciseness. Note that this result is consistent with ERP studies which investigated the emotional Stroop task in non-clinical participants and did not report effects on the EPN component (e.g., Li et al., 2007; van Hooff et al., 2008; Sass et al., 2010).

^4^Our aim was to observe whether adjectives produced a larger Stroop effect than substantives but the results showed no behavioral and electrophysiological differences. Therefore, we did not distinguish these two grammatical classes in our analyses.

^5^The PCL-C test indicates the presence of a significant level of PTSD symptom severity for scores superior or equal to 50. No one victim had a score superior or equal to 50 in this study. Moreover, no one participant reported have been diagnosed PTSD.

## Appendix

**Table:** Number of participants as a function of group (PTSD, control) for EEG studies with samples smaller or equal to those of the present study.

	***N* PTSD group**	***N* control group (exposed or non-exposed)**
Araki et al., 2005	8	13
Blomhoff et al., 1998	11	9
Hunter et al., 2011	7	11
Kounios et al., 1997	8	8
Lamprecht et al., 2004	10	10
Menning et al., 2008	10	14
Metzger et al., 1997	9	10
Stanford et al., 2001	10	10
Weber et al., 2005	10	10
Wessa et al., 2005	7	7
Yun et al., 2011	12	12
Zhang et al., 2014	13	13
